# Bronchoalveolar Lavage Fluid Utilized Ex Vivo to Validate In Vivo Findings: Inhibition of Gap Junction Activity in Lung Tumor Promotion is Toll-Like Receptor 4-Dependent

**DOI:** 10.4172/2155-9929.1000160

**Published:** 2013-12-27

**Authors:** Thomas Hill, Ross S Osgood, Kalpana Velmurugan, Carla-Maria Alexander, Brad L Upham, Alison K Bauer

**Affiliations:** 1 Department of Environmental and Occupational Health, Colorado School of Public Health, University of Colorado, Aurora, Colorado, USA; 2 Department of Pediatrics and Human Development, Michigan State University, Lansing, USA

**Keywords:** Bronchoalveolar Lavage Fluid (BALF), Butylated Hydroxytoluene (BHT), Connexin 43 (cx43), Gap junctions, Gap Junction Intercellular Communication (GJIC), Lung, Toll-like receptor 4 (TLR4), Tumor Promotion

## Abstract

TLR4 protects against lung tumor promotion and pulmonary inflammation in mice. Connexin 43 (Cx43), a gap junction gene, was increased in *Tlr4* wildtype compared to *Tlr4*-mutant mice in response to promotion, which suggests gap junctional intercellular communication (GJIC) may be compromised. We hypothesized that the early tumor microenvironment, represented by Bronchoalveolar Lavage Fluid (BALF) from Butylated hydroxytoluene (BHT; promoter)-treated mice, would produce TLR4-dependent changes in pulmonary epithelium, including dysregulation of GJIC in the *Tlr4*-mutant (BALB^*Lps-d*^) compared to the *Tlr4*-sufficient (BALB; wildtype) mice. BHT (4 weekly doses) was injected ip followed by BALF collection at 24 h. BALF total protein and total macrophages were significantly elevated in BHT-treated BALB^*Lps-d*^ over BALB mice, similar to previous findings. BALF was then utilized in an *ex vivo* manner to treat C10 cells, a murine alveolar type II cell line, followed by the scrape-load dye transfer assay (GJIC), Cx43 immunostaining, and quantitative RT-PCR (*Mcp-1, monocyte chemotactic protein 1*). GJIC was markedly reduced in C10 cells treated with BHT-treated BALB^*Lps-d*^ BALF for 4 and 24 h compared to BALB and control BALF from the respective mice (p < 0.05). *Mcp-1*, a chemokine, was also significantly increased in the BHT-treated BALB^*Lps-d*^ BALF compared to the BALB mice, and Cx43 protein expression in the cell membrane altered. These novel findings suggest signaling from the BALF milieu is involved in GJIC dysregulation associated with promotion and links gap junctions to pulmonary TLR4 protection in a novel *ex vivo* model that could assist in future potential tumor promoter screening.

## Introduction

Lung cancer is a largely preventable disease in the U.S. and abroad [1]. Lung tumor formation is often a 2-stage process, starting with initiation, a direct mutagenic event, followed by promotion, a nongenotoxic event leading to the clonal expansion of initiated cells and tumor development. A common 2-stage model in the lung uses 3-Methylcholanthrene (3-MCA) followed by sequential dosing of Butylated Hydroxytoluene (BHT) in mice [2]. In more recent and past studies, the inflammatory characteristics of Bronchoalveolar Lavage Fluid (BALF) from treated mice have been used to describe changes in the local microenvironment that may be predictive of tumorigenesis [3–6].We previously demonstrated that functional Toll-Like Receptor 4 (TLR4), an innate immune receptor, is protective against inflammation and primary tumor formation in the mouse lung *in vivo* using the MCA/BHT 2-stage promotion model [3,7].

TLR4 both exacerbates and protects from inflammation and injury in pulmonary models. For example, TLR4 exacerbates lipopolysaccharide (LPS)-induced lung injury [8]. However, TLR4 protects against lung infection and other pulmonary diseases, such as emphysema [9,10]. In epidemiological studies, farm and textile workers had significant associations with decreased lung cancer risk in those individuals exposed to endotoxin (LPS) [11]. The primary receptor that binds endotoxin is TLR4 [8], therefore, it is likely involved in the protection observed with endotoxin exposure. Additionally, TLR4 confers protection in other organ systems, such as human gastric carcinomas [12].

Gap junctions are intercellular channels composed of connexin (cx) protein hexamers that permit cellular communication between neighboring cells and have been shown to regulate signal transduction pathways [13]. Impaired function of gap junctions secondary to toxicant exposure is strongly associated with pathological states, such as cancer and developmental defects [13,14]. Few studies have assessed pulmonary gap junction functionality, thus we have little understanding of the role(s) of gap junctions in pulmonary disease [15,16]. The scrape load dye-transfer (SL/DT) assay, a well-established *in vitro* assay [17] is commonly used to measure gap junction intracellular communication (GJIC). As such, GJIC has potential as a mechanistic biomarker to elucidate cellular signaling events involved in carcinogenesis that are not readily achievable in an *in vivo* system. Our previous transcriptomics study of TLR4-dependent effects in the mouse lung identified connexin 43 (Cx43), the major lung gap junction protein, as a gene potentially altered during early tumorigenesis [7].While both the BHT promotion model and the SL/DT assay have been used independently to evaluate carcinogenic or toxic potential *in vivo* and *in vitro*, the coordinated use of these two tests as an *ex vivo* method to mechanistically examine the effects of tumor promoters is a novel adaptation with potential for both translational studies and clinical applications.

Most of the published literature to date has focused on the effects of complete carcinogens and mixtures of initiators and promoters on carcinogenesis (e.g. tobacco smoke), with little work directed at the promotion stage itself, which is the reversible stage of tumor development [2,18]. Examples of potential promoters include a long list of toxicants, such as polycyclic aromatic hydrocarbons that are known environmental contaminants attributable to combustion (fossil fuels, smoking) [16,19], and other pollutants such as Vanadium Pentoxide (V_2_O_5_) [5]. The alteration of the inflammatory profile in the local microenvironment by such tumor promoting compounds (eg. BHT, PAHs, V_2_O_5_, or 12-O-tetradecanoylphorbol-13-acetate (TPA)) is a proposed mechanism for the actual events leading to tumor formation and progression [4,16,20]. If correct, sentinel events such as dysregulation of GJIC, should have some link to immune signaling within the cells, consistent with our *in vivo* findings for enhancement of inflammation and tumorigenesis in those mice with a mutation in *Tlr4*, as previously noted. In our studies, *we hypothesized that the effects of the early tumor microenvironment, represented by BALF from BHT-treated mice, would produce TLR4-dependent changes in cultured pulmonary epithelium that will include differential inhibition of GJIC*. To do so, we measured GJIC and various inflammatory markers in a murine pulmonary epithelial cell line (C10 cells) treated with BALF from BHT-exposed mice or controls to discretely evaluate the existence of a TLR4-dependent response to a known lung tumor promoter.

## Materials and Methods

### Animal studies and Bronchoalveolar Lavage Fluid (BALF) processing

The mice used were age matched 5–7 week old male BALB/c (BALB; *Tlr4* sufficient) from Jackson Laboratories and C.C3H-*Tlr4^Lps-d^* (BALB^*Lpsd*^, *Tlr4* mutant) mice bred in the CU animal facility. The BALB^*Lpsd*^ mice contain a mutant dominant negative *Tlr4* that is not functional [21]. The animals were housed in the University of Colorado Animal Care Facility under environmentally controlled conditions and veterinary supervision. They were fed irradiated mouse chow (Harlan) and water *ad libitum*, and no pesticides or chemicals were used in the animal rooms for the duration of the experiments. All animal use was approved by the University of Colorado Denver Institutional Animal Care and Use Committee and follows the Helsinki convention for the use and care of animals.

BALB and BALB^*Lps*^-d mice were administered intraperitoneal (ip) injections of BHT (Sigma, St. Louis, MO) dissolved in corn oil (vehicle control) once per week for 4 wks(150 (wk 1), 200 mg/kg (wks 2–4)), similar to previous studies [3,4,22]. Twenty-four hours after the final BHT dose, the mice were euthanized with Sleepaway (120 mg/kg; MWI, Boise, ID) and four bronchoalveolar Lavages performed using Hanks buffered salt solution (HBSS) at 35 ml/kg body weight. The initial lavage was reserved, centrifuged, and the cell-free supernatant frozen at −80°C for use on the C10 cells after an aliquot was removed for total protein analysis (BioRad, Hercules, CA) following previous studies [3,5,7,23,24]. Total BAL protein is an indicator of lung hyperpermeability [25]. Cell pellets from all lavages were combined per mouse and total cells counted, followed by cytocentrifugation and Diffquick staining (Sigma, St. Louis, MO) for cell differentials using previously established methods [3,5,7,23,24].

### *In vitro* studies

A murine alveolar Type II cell line derived from BALB/c mice (C10 cells) was used, which was a kind gift from Dr. Lori Dwyer-Nield (University of Colorado) and originates from Smith et al. (1985) [26]. Many studies have used the C10 cells as a surrogate cell to mimic the type II cells in the lung in response to several toxicants and they have been well characterized [16,27,28]. The cells were maintained in confluent culture conditions with CMRL (Invitrogen, Grand Island, N.Y.) in 10% fetal bovine serum (FBS, Atlanta Biologicals; Atlanta, GA) medium supplemented with glutamine at 37°C and 5% CO_2_.

**Figure 1:**
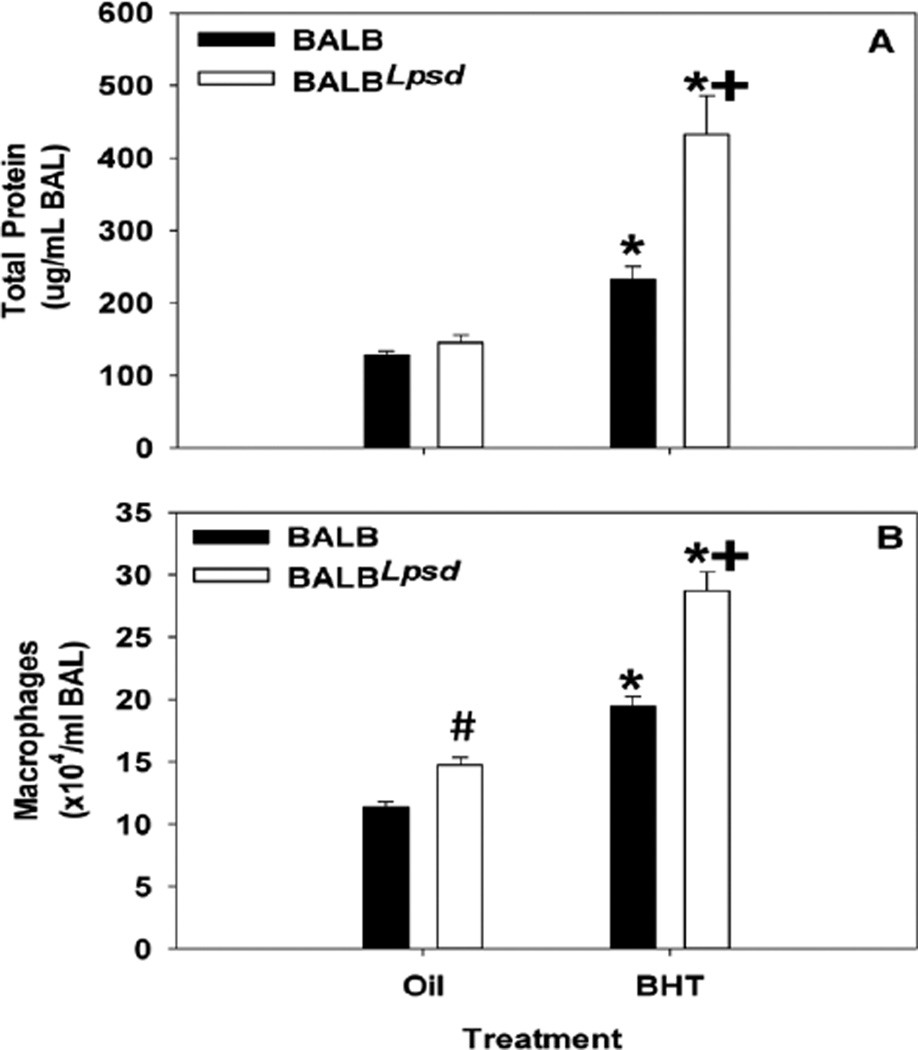
BHT produces an inflammatory profile in treated mice. (A) Total protein content was increased in both strains in response to BHT, however was significantly higher in BHT treated BALB^*Lps-d*^ mice as indicated by the plus sign(N=5; p < 0.001). (B) Total macrophage infiltration of BALF in BALB^*Lps-d*^ mice is statistically significant for all treatment groups (N=5; p < 0.05). Data presented as mean and SEM. *, P<0.05 for BHT compared to oil treatment groups; +, p<0.05 for BALB versus BALB^*Lps-d*^ mice; #, p<0.05 for oil treatment between strains.

For all *ex vivo* studies, C10 cells were grown to confluence over 2–3 days in 12 well dishes or on coverslips (immunocytochemistry). At confluence, the cells were switched to serum-free medium for 24 h. The wells were then treated with BALF from individual mice diluted 1:4 in serum-free medium for 4 and 24 h. Figure 1A demonstrates differences in protein concentration between the strains. TPA, a liver tumor promoter and a known inhibitor of GJIC [29–32], was used as a treatment control along with serum-free medium.

### Scrape-load/dye-transfer (SL/DT) assay

At 4 and 24 h, GJIC was assessed using the *SL/DT* assay, as previously reported [16,17,29]. The intact cell monolayer was rinsed 3 times with PBS, followed by application of Lucifer Yellow dye (1% in PBS; Sigma) to the wells. Cuts were made in the monolayer with a surgical scalpel, and the cells were incubated for 3 min at room temperature. The wells were then rinsed 3 times with PBS and fixed with 4% formalin. A Nikon Eclipse Ti fluorescent microscope system (Nikon Instruments Inc.; Melville, NY) was used to capture images followed by Image J software (ImageJ; NIH) for quantitation of the digitized images. Calculations of GJIC were performed according to the published method [17].

### RNA isolation and real-time quantitative reverse transcriptase-polymerasechainreaction (qRT-PCR)

After exposure to BALF for 24 h, RNA was extracted from C10 cells using the Nucleospin RNA II kit according to the manufacturer’s protocol (Macherey-Nagel,Düren, Germany).Whole lung RNA was extracted using the RNAeasy miniprep kit (Qiagen, Germantown, Maryland). For qRT-PCR, 1 µg of total RNA from each sample was converted to cDNA using oligo-dT, followed by PCR amplification in 50 µL reaction volumes. The cDNA stock was used undiluted for qRT-PCR using gene specific, intron-spanning primers. *Mcp-1*primers:[1] 5’-GTCACCAAGCTCAAGAGAGA-3’, (Rev) 5’-GTCACTCCTACAGAAGTGCT-3; *Gja1* (*Cx43*) primers [1] 5’-CAACGTGGAGATGCACCTGAAG-3’, (Rev) 5’-GCACTCAGGCTGA ACCCATAGA-3’. Primers for *Cox-2* and *iNOS* were previously published in Osgood et al. 2013 [16] and Buxade et al. 2012 [33]. All *Mcp1, Cox-2, and iNOS* reactions were carried out in a final volume of 25µL using KAPA SYBR® FAST master mix (Kapa Biosystems; Boston, MA) on a Master cycler EP Realplex_4_ qRT-PCR cycler (Eppendorf; Hauppauge, NY). *Cx43* reactions were carried out similar to those in Bauer et al. 2011 [23] using Sybr Green (Applied Biosystems, Foster City, CA) and an ABI Prism 7700 Sequence Detection System (Applied Biosystems). Reactions were carried out in triplicate for each sample. Ct values for gene induction were normalized to those of 18S as previously described [7], and calculations of fold induction determined [34]. All graphics are representative of the results from at least three independent experiments.

### Immunocytochemical staining

After 4 and 24 h BALF exposures, the cells were fixed with 4% paraformaldehyde for 30 minutes. After washing in PBS, the cells were permeabilized, and blocked with 5% normal goat serum and 0.2% Triton X-100 in PBS for 90 min in a humidified chamber. Cells were then exposed to rabbit polyclonal Cx43 primary antibody (Cell Signaling, Danvers, MA) at a dilution of 1:150 in 3% Bovine Serum Albumin (BSA) and incubated at 4°C overnight. The coverslips were again washed followed by incubation for 90 min at room temperature in the dark with the secondary antibody, Alexa Fluor 488-conjugated anti-rabbit IgG (1:250) (Invitrogen). After washing, coverslips were coated with Prolong Gold anti-fade mounting medium containing DAPI (Invitrogen), allowed to dry overnight, then sealed and imaged on a Nikon D-Eclipse C1 confocal microscope (Nikon Instruments Inc.; Melville, NY).

### Statistical Analysis

The following statistical analyses were used to assess macrophage infiltration, total BALF protein, SL/DT assay and qRT-PCR: 2-way ANOVA with factors (strain X treatment) and a Student Newman Keuls post hoc comparison of relevant groups, using SigmaPlot (Systat Software; Chicago, IL).

## Results

### Characterization of the inflammatory state of the BALF

We demonstrated an increase in total BALF protein content and macrophage infiltration in all mice exposed to BHT (Figure 1A and 1B), similar to previous studies [3], for comparison to the analysis of gap junction activity in the experiments that followed (Figures 3 and 4). Total BALF protein significantly increased in both strains in response to BHT compared to oil controls (p≥0.02), however the BALB^*Lps-d*^ mice had significant elevations above that observed in the BALB BHT-treated mice (Figure 1A). Additionally, the magnitude of macrophage infiltration in the BALF of BALB^*Lps-d*^ mice was greater than that of the BALB, in response to BHT (Figure 1B). These data clearly show that an active inflammatory state was induced in all exposed animals, and that the collected BALF should therefore reflect the character of the local pulmonary microenvironment after exposure to a known promoter.

### GJIC is altered in C10 cells following treatment with BALF from BALB^Lps-d^ mice

**Figure 2:**
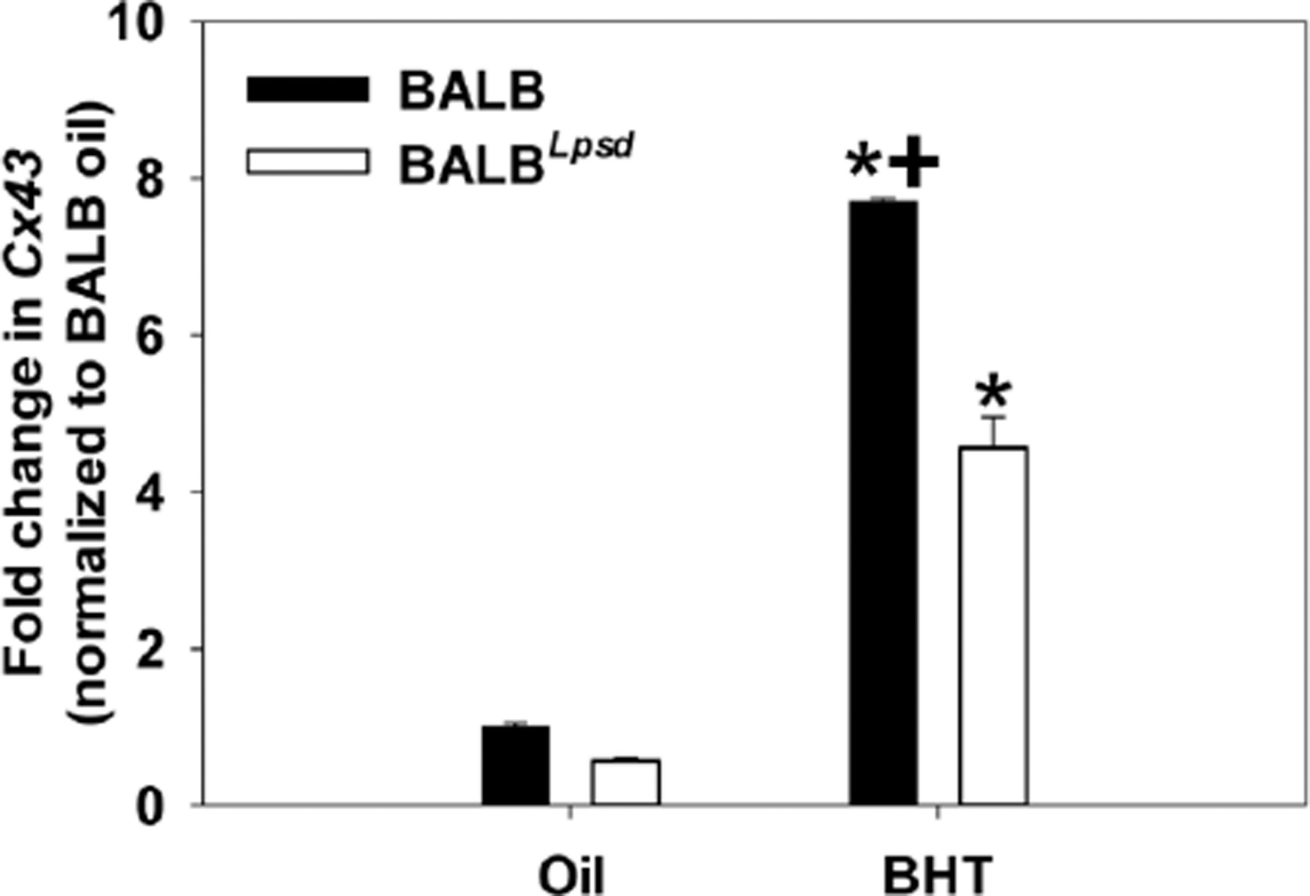
*Cx43* mRNA expression in whole lung 24 h following in subchronic treatment of mice with BHT. Cx43 was first normalized to 18S followed by normalization to the BALB oil control, as done previously [7]. Mean and SEM are presented of fold-change in *Cx43* (n =3 mice per treatment; repeated three times).*, P<0.05 for BHT compared to oil treatment groups; +, p<0.05 for BALB versus BALB^*Lps-d*^ mice.

**Figure 3:**
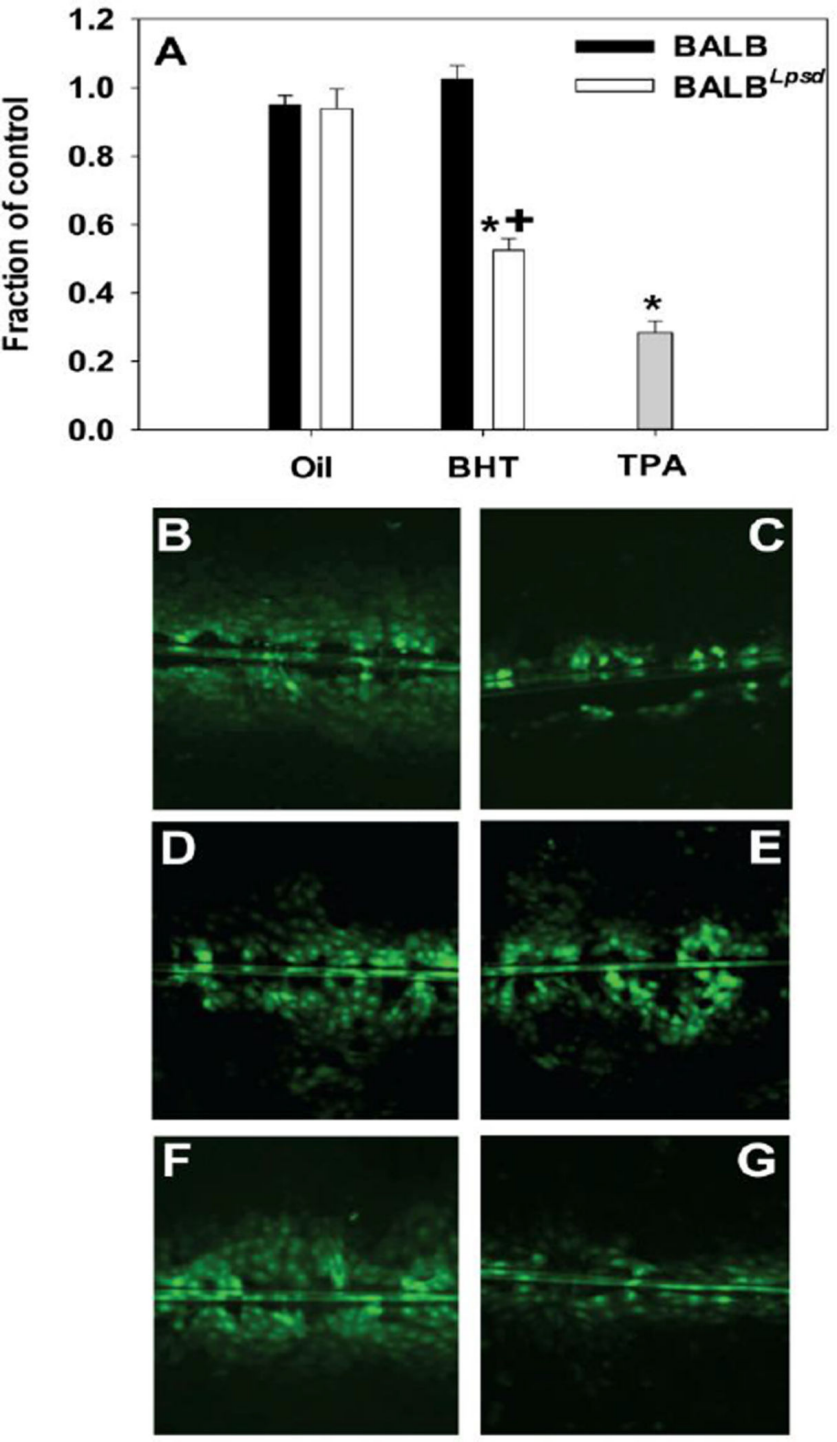
Inhibition of GJIC after a 4 h exposure to BALF is both treatment and strain dependent. A) Graph of quantitated images from both BALB and BALB^*Lps-d*^ BALF-treated C10 cells following the SL/DT assay using Lucifer Yellow fluorescent dye. N=4–7 per treatment group, repeated twice. Data were normalized to the medium control and presented as mean + SEM for fraction of control. *, P<0.05 for BHT compared to oil treatment groups; +, p<0.05 for BALB versus BALB^*Lps-d*^ mice. More inhibition is evident in cells treated with BALF from BHT-treated BALB^*Lps-d*^ (G) compared to all other BALF treatments. (B) C10 cells treated with serum-deprived media alone as control; (C) C10 cells treated with TPA as a known inhibitor of GJIC. (D) C10 cells treated with BALF from oil-treated BALB mice; (E) C10 cells treated with BALF from BHT-treated BALB mice; (F) C10 cells treated with BALF from oil-treated BALB^*Lps-d*^ mice; (G) C10 cells treated with BALF from BHT-treated BALB^*Lps-d*^ mice. Magnification is 100×.

**Figure 4:**
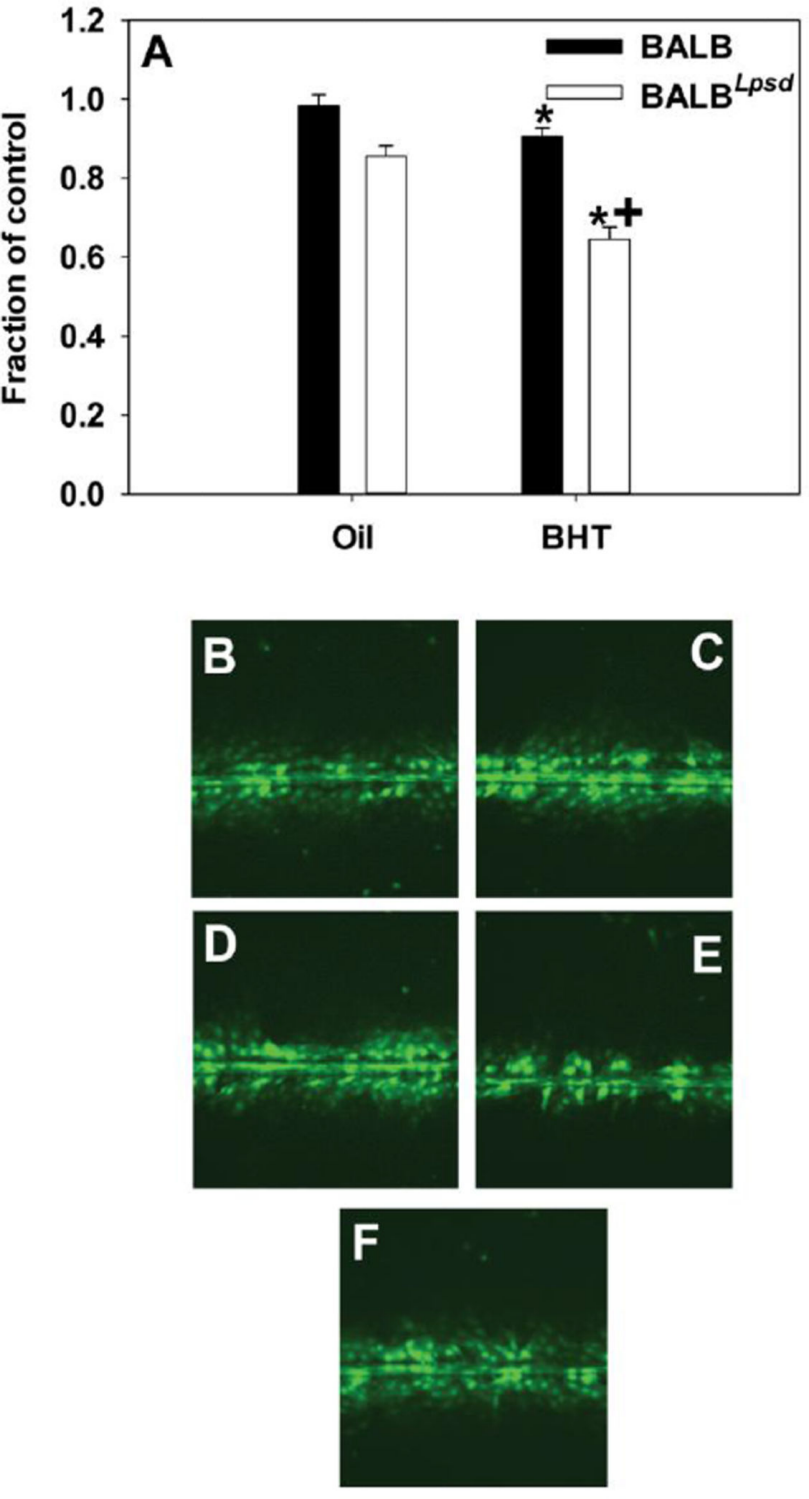
Inhibition of GJIC after a 24 h exposure to BALF is both treatment and strain dependent. (A) Quantitation of SL/DT assay demonstrating significant differences between strain BALF effects in the C10 cells following the SL/DT assay using Lucifer Yellow fluorescent dye. N=3–5 per treatment group, repeated twice. Data were normalized to the medium control and presented as mean + SEM for fraction of control. *, P<0.05 for BHT compared to oil treatment groups; +, p<0.05 for BALB versus BALB^*Lps-d*^ mice. (B) Image of C10 cells treated with BALF from oil-treated BALB mouse; (C) Image of of C10 cells treated with BALF from BHT-treated BALB mouse; (D) Image of C10 cells treated with BALF from oil-treated BALB^*Lps-d*^ mouse; (E) Image of C10 cells treated with BALF from BHT-treated BALB^*Lps-d*^ mouse; (F) Image from C10 cells treated with serum-deprived media alone as control. Magnification is 100×.

*Cx43* gene expression was reduced by BHT exposure in the lungs of BALB^*Lps-d*^ mice compared to BALB mice (Figure 2) suggesting impaired gap junction signaling. In an effort to determine the actual effects on GJIC activity, we assessed the effects of BALF from treated and control mice on GJIC in C10 cells. After treatment with BALF for 4 h, gap junctions in the C10 cells were dysregulated in a manner consistent with both strain and treatment characteristics of the BALF donor (Figure 3). GJIC activity was reduced in response to BALF from BALB^*Lps-d*^ mice and approached the degree of inhibition produced by the treatment control, TPA. Following 24 h treatment with the BALF, we observed similar inhibition of GJIC in the BALB^*Lps-d*^ mice, although the magnitude of response was lower than that observed at 4 h (Figure 4). No statistically significant reduction in GJIC signaling was observed in the C10 cells following application of BALF from BALB mice regardless of treatment group or duration of exposure. These results indicate that Tlr4 protects cells from the disruption of GJIC by BHT induced release of inflammatory factors. The biphasic inhibition of GJIC by TPA peaks at 30 min and recovers at 24 h, thus it was only used at 4 h [29].

### GJIC inhibition correlates with redistribution of Cx43 protein but not Cx43 expression

**Figure 5:**
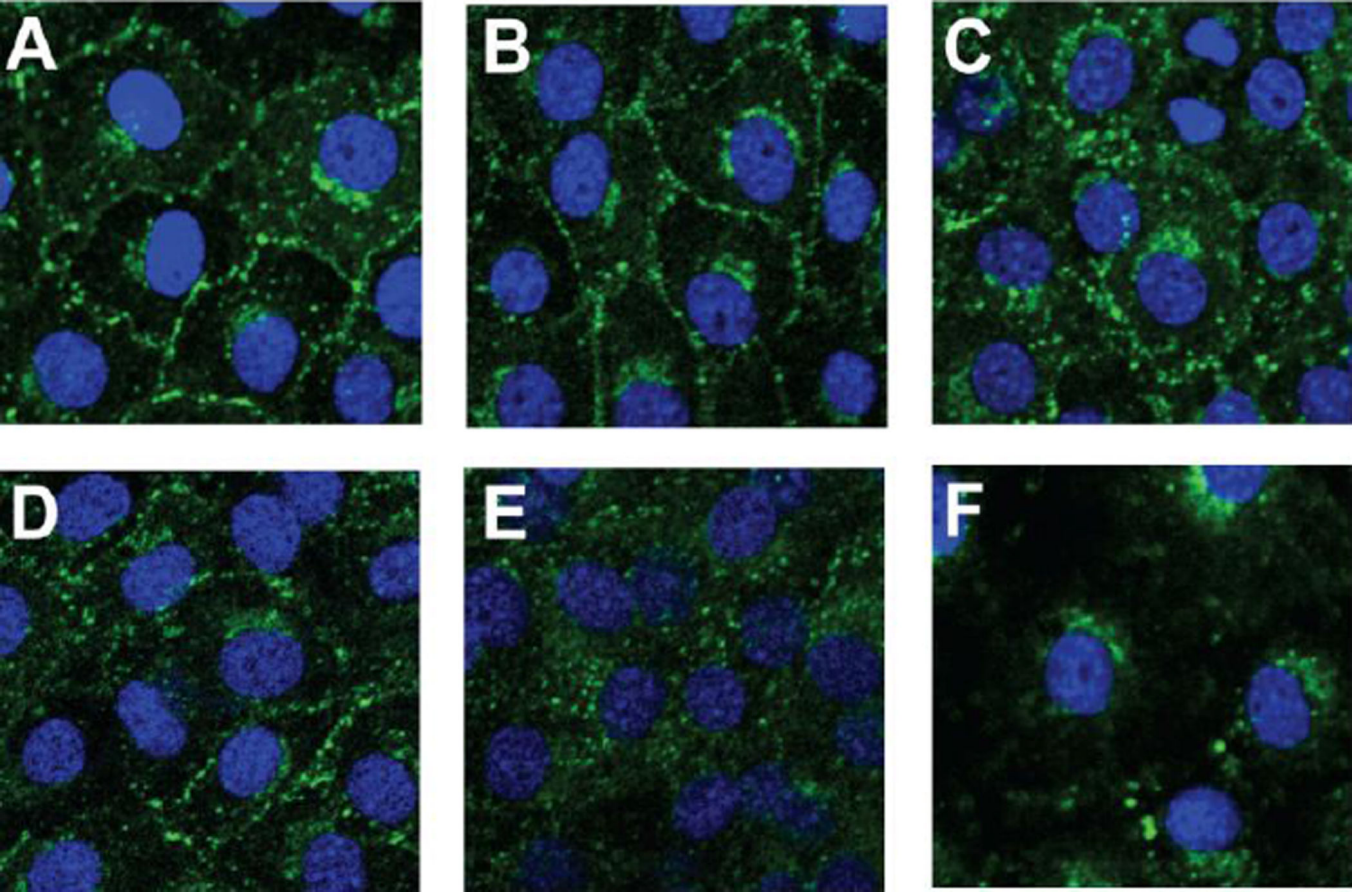
Connexin 43 membrane localization is altered in BALB^*Lpsd*^ mice. Cx43 immunostaining in C10 cells exposed to BALF from BALB and BALB^*Lpsd*^ mice for 4 h. DAPI was used as the nuclear stain and Alexa-Fluor 488 linked to specific Cx43 antibodies. (A) C10 cells treated with serum-deprived media as the control; (B) C10 cells treated with BALF from BALB oil-treated mice; (C) C10 cells treated with BALF from BALB BHT-treated mice; (D) C10 cells treated with BALF from BALB^*Lpsd*^ oil-treated mice; (E) C10 cells treated with BALF from BALB^*Lpsd*^ BHT-treated mice; (F) C10 cells treated with TPA as a tumor promoter control to demonstrate removal of Cx43, as observed previously [16,29]. N=2–3 for each treatment group and repeated twice. Magnification, 1000×.

While GJIC was altered in response to BALF from BHT-treated mice, neither total protein levels or phosphorylation patterns of Cx43 were altered in the C10 cells at either 4 or 24 h (immunoblotting, data not shown). To determine if the observed inhibition of GJIC might be related to physical alteration of connexons, subcellular localization patterns of Cx43 within the cells was assessed using immunostaining (Figure 5). C10 cells treated with BALF from oil-treated mice did not affect the distribution patterns of Cx43 on the plasma membrane (Figure 5B, 5D) while cells treated with BALF collected from BALB mice exposed to BHT displayed an increase in cytosolic localization of Cx43 but retained abundant Cx43 expression at the plasma membrane (Figure 5C). Cells treated with BALF collected from the BHT-treated BALB^*Lps-d*^ mice displayed significant translocation of Cx43 from the plasma membrane to the cytosol at 4 h of exposure (Figure 5E). This effect was not seen in the C10 cells after a 24 h exposure period (data not shown) in spite of ongoing GJIC inhibition, suggesting that new connexin protein was synthesized, but failed to fully restore the connexon and regain normal function.

### Molecular marker of inflammation

**Figure 6:**
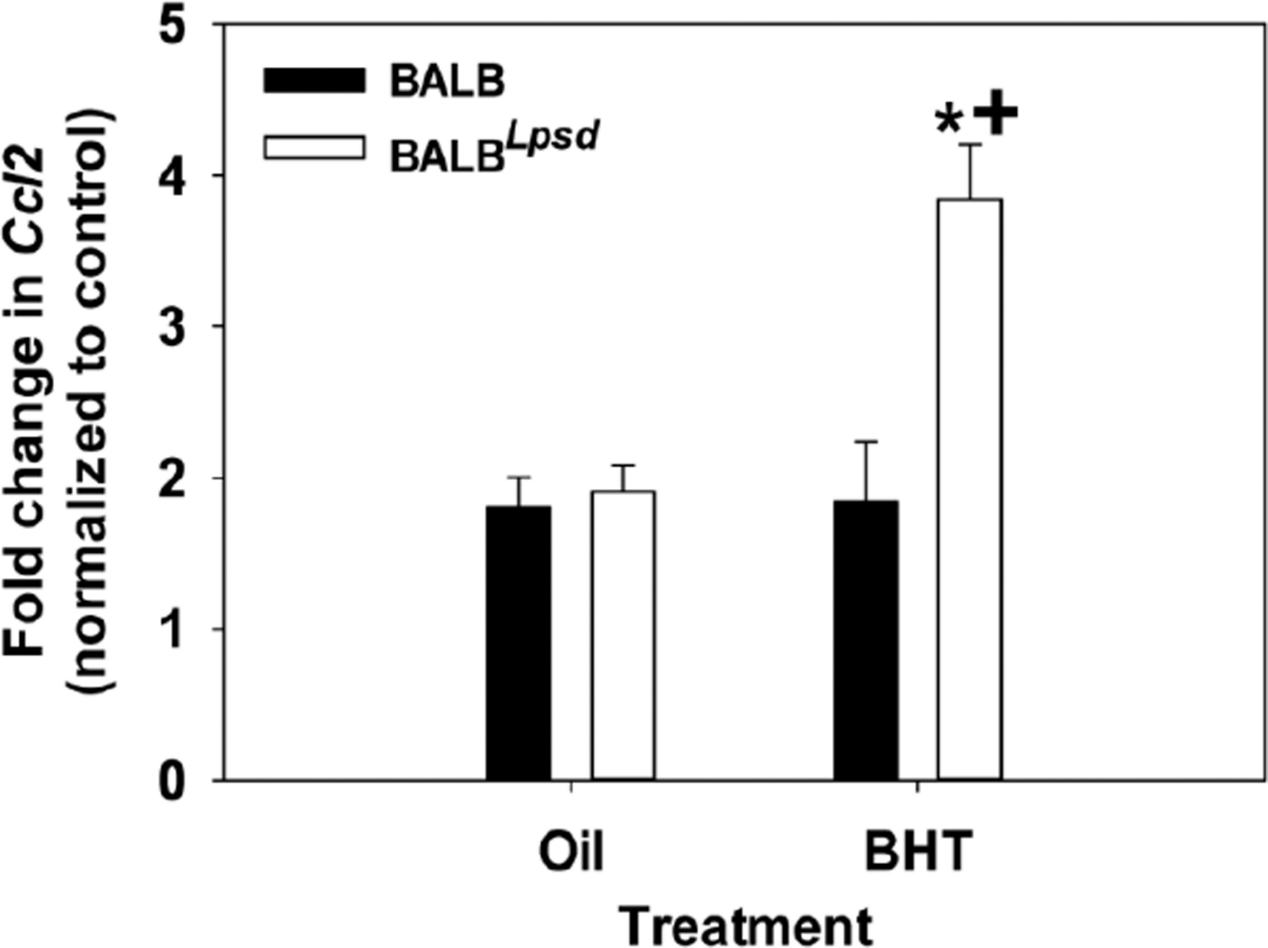
BALF treatment causes differential induction of an innate immune marker *in vitro*. A) C10 cells display higher levels of chemokine ligand 2 (*Ccl2; Mcp-1*) mRNA induction when exposed to BALF from BHT-treated BALB^*Lps-d*^ mice as opposed to BALB mice. Data were normalized to the medium control and are presented as mean fold induction and SEM; N=5 for each treatment group.

Monocyte Chemotactic Protein 1 (MCP-1 or CCL2), a chemokine that is a macrophage/monocyte chemoattractant, was previously identified in a transcriptomic study as a pathway involved in responsiveness to BHT in the BALB^*Lps-d*^ mice compared to BALB mice [7]. We observed significant increases in *Mcp-1* mRNA expression in C10 cells treated with BALF collected from the BALB^*Lps-d*^ mice exposed to BHT as compared to cells treated with BALF from the BHT-treated BALB mice and controls (Figure 6). We also evaluated *Cox-2* and *iNOS* mRNA expression in the same manner as for *Mcp-1*, but did not observe any significant changes among these inflammatory markers in response to the *ex vivo* BALF administration with either strain (data not shown).

## Discussion

In our study, we evaluated TLR4 dependence of GJIC during alterations in the local microenvironment produced by the promoter BHT. To do so, we exposed both BALB (*Tlr4* sufficient) and BALB^*Lps-d*^ (*Tlr4* mutant) mice to BHT and collected their BALF. Strain or treatment differences in the cell-free BALF were documented and further characterized *ex vivo* using C10 cells treated with BALF. Our findings demonstrate that exposure of C10 cells to BALF from mice exposed to BHT produces unique *ex vivo* treatment and strain-based effects on cellular gap junctions that are TLR4-dependent. Interestingly, resveratrol, a phytoalexin present in grapes can reverse the GJIC-inhibitory effects of promoters [35], and these chemopreventative actions in cutaneous carcinomas appear to be TLR4-dependent [36].

Due to the limited metabolic capacity of the C10 cell line [37], it is likely that a xenobiotic or endogenous compound(s) already present in the BALF are responsible for the dysregulation of GJIC in C10 cells at 4 and 24 h. The significant difference in the total protein content of the BALF from the exposed BALB^*Lps-d*^ mice, combined with the known rapid clearance of BHT from previously treated animals [38] suggests this may be protein-linked. The dependence of this dysregulation on a compromised TLR4 response and the magnitude of macrophage infiltration in BALB^*Lps-d*^ mice, suggests that the responsible mediator may be part of an immune signaling cascade. In addition, *Mcp-1* expression was induced in treated C10 cells, which are in parallel with the BALF macrophage response to BHT *in vivo*.

There is substantial supporting evidence that lung tumor promotion, particularly using the BHT model, involves inflammation [4,5,7,25,39–43]. We refer the readers to several reviews on inflammation and lung cancer for more mechanistic details, as it is not the primary focus of these studies [20,22,44–46]. However, we and others have previously demonstrated using the BHT model that specific inflammatory cell types (ie. macrophages, lymphocytes, and neutrophils) are likely eliciting a cytokine/chemokine or other inflammatory mediator (ie. prostaglandins) response that can further lead to proliferation of initiated epithelial cells to advance the tumorigenic process [3,4,25,40,43,47]. Our current studies implicate inflammation as a major response to BHT, thus this BHT-induced tumor model will be expanded upon by further characterization of inflammatory cell types, such as lymphocyte phenotypes, determination of cytokine/chemokine profile responses, as well as identify involvement of downstream growth factor pathways. Some evidence, including our previous *in vitro* studies in C10 cells [16], supports a role for a cytokine associated inhibition of gap junctions. Whether this is a direct or indirect effect and the timing of this effect (acute vs chronic), needs to be further investigated using methods such as recombinant cytokine/chemokine administration or inhibition of cytokine/chemokine pathways using antibodies or siRNA techniques followed by evaluation of gap junctions. Thus far, one study in human airway epithelial cells using recombinant tumor necrosis factor α (TNF) demonstrated a direct inhibitory effect on gap junctions [48]. Future studies will investigate alterations in gap junction activity, using approaches such as those described above, that are both inflammation-dependent and independent, as well as related mechanisms involved in altering gap junction activity and those inflammatory events that are unrelated to GJIC.

Connexin 43 (Cx43) is a component of gap junctions that are expressed in the lung in multiple cell types, including both alveolar type II cells and bronchiolar Clara cells [13]. Type II cells and Clara cells are the progenitor cells for lung adenocarcinoma and are hence often studied as primary cellular targets for toxicants, such as tumor promoters [49–51]. Cx43 is the predominant pulmonary connexin, and is present and active in C10 cells [13,27]. *Cx43* was significantly reduced in response to direct application of BHT (parent compound effect) in *in vitro* studies [27] and mice heterozygous for mutant *Cx43* are more susceptible to tumor development in a urethane tumorigenesis model [52]. Aberrant *CX43* expression has also been reported in biopsies from in NSCLC patients [53]. Our immunoblots for Cx43 did not demonstrate differences in protein content between or within treatment groups. This suggests that the observed inhibition of GJIC might be due to a functional change in the connexins rather than simply protein ubiquitination and degradation, as has been reported with the direct use of toxicants on these cells in our laboratory [16]. Since the localization appears to change from membrane to cytosol in response to the BALF (Figure 5), we propose that this movement is a cell-directed reshuffling event which attempts to reorganize connexins back into functional gap junctions, as suggested by the incomplete return of gap junctional activity by 24 h compared to 4 h. Cx43 in functional gap junctions is known to display multiple phosphorylated forms in immunoblots. While we observed no changes in Cx43 phosphorylation patterns using several antibodies (Chemicon, Cell Signaling), it is possible that as yet undiscovered Cx43 phosphorylation sites could demonstrate phosphorylation differences after BALF exposure.

While the traditional method of direct toxicant exposure to cells *in vitro* has produced valid data in the past, the use of Bronchoalveolar Lavage Fluid (BALF) from exposed animals offers several advantages that make it a more thorough and useful approach to elucidate the cellular mechanisms in the many stages of carcinogenesis. First, it eliminates the issue of limited or unknown metabolic capacity in the pure *in vitro* system. By using biological fluids from an intact organism, the complete *in vivo* metabolite profile for the test compound is brought to bear upon the cultured cells, which greatly increases the potential for detection of toxicant effects. It also reduces the risk of false hits from the parent compound which may not be present at the target tissue site *in vivo*. Secondly, it has the possibility of expanding the translational capacity for mechanistic research into the stages of carcinogenesis in the human population. In this system, the use of biological fluids (eg. BALF) from human subjects could potentially be used as an evaluation tool (a biomarker) for exposure to cancer promoters. In addition, the system can determine cellular response patterns between *in vitro* models in human cell lines and lung cancer patients in both early and late stage pathologies. These potential comparisons would not only permit enhanced mechanistic explanation of the early, reversible stages of cancer development in humans, it may also present an enticing format for high-throughput evaluation of chemotherapeutic interventions as well.

Using this novel system we demonstrated marked inhibition of GJIC and alterations in *Mcp-1* transcription patterns in C10 cells that are both treatment and strain dependent. These *in vitro* findings are in parallel with the inflammatory profile of the BALF collected from donor mice that were exposed *in vivo*. These novel findings suggest endocrine signaling from the inflammatory milieu are involved in the dysregulation of GJIC during the promotion phase of carcinogenesis. Furthermore, these data establish a link between dysregulation of gap junctions, tumor promotion, and the influence of TLR4 in an *ex vivo* model which will facilitate a mechanistic examination of these protective effects at the molecular level.
